# In order for the light to shine so brightly, the darkness must be present—why do cancers fluoresce with 5-aminolaevulinic acid?

**DOI:** 10.1038/s41416-019-0516-4

**Published:** 2019-08-13

**Authors:** Kym McNicholas, Melanie N. MacGregor, Jonathan M. Gleadle

**Affiliations:** 1 Department of Renal Medicine, Flinders Medical Centre, Flinders University, Bedford Park, SA 5042 Australia; 2 College of Medicine and Public Health, Flinders University, Bedford Park, SA 5042 Australia; 3 Future Industries Institute, School of Engineering, University of South Australia, Adelaide, SA 5095 Australia

**Keywords:** Cancer imaging, Cancer metabolism, Cancer microenvironment

## Abstract

Photodynamic diagnosis and therapy have emerged as a promising tool in oncology. Using the visible fluorescence from photosensitisers excited by light, clinicians can both identify and treat tumour cells in situ. Protoporphyrin IX, produced in the penultimate step of the haem synthesis pathway, is a naturally occurring photosensitiser that visibly fluoresces when exposed to light. This fluorescence is enhanced considerably by the exogenous administration of the substrate 5-aminolaevulinic acid (5-ALA). Significantly, 5-ALA-induced protoporphyrin IX accumulates preferentially in cancer cells, and this enhanced fluorescence has been harnessed for the detection and photodynamic treatment of brain, skin and bladder tumours. However, surprisingly little is known about the mechanistic basis for this phenomenon. This review focuses on alterations in the haem pathway in cancer and considers the unique features of the cancer environment, such as altered glucose metabolism, oncogenic mutations and hypoxia, and their potential effects on the protoporphyrin IX phenomenon. A better understanding of why cancer cells fluoresce with 5-ALA would improve its use in cancer diagnostics and therapies.

## Background

In 1903, Niels Ryberg Finsen was awarded the Nobel Prize in Medicine “in recognition of his contribution to the treatment of diseases … with concentrated light radiation, whereby he has opened a new avenue for medical science”.^1^ Twenty years later, in 1924, Policard observed selective fluorescence by porphyrins in rat sarcoma tissue.^2^ Since then, a variety of photosensitiser drugs that detect tumours and induce targeted cell damage in response to light have been investigated and tested on different tumours, launching the fields now known as photodynamic diagnosis (PDD) and photodynamic therapy (PDT). PDD uses the targeted delivery of light at the appropriate wavelength to excite a photosensitiser to emit fluorescence at the tumour site. With sufficient light intensity, the resultant photochemical reaction is thought to induce reactive oxygen species (ROS)-mediated cell death in the process known as PDT.

The first photosensitiser prodrug to gain worldwide clinical approval was a hematoporphyrin derivative called Porfimer sodium (Photofrin^®^) in 1993. Modern photodynamic diagnostic approaches, such as those used for the tumour resection of bladder^3^ and brain cancers,^4^ now rely on the distinctive fluorescence of malignant and premalignant lesions induced by the exogenous administration of a photosensitiser or its precursor. Successful treatment of cancers including skin and lung cancers alongside more than 50 ongoing clinical trials using 11 different photosensitisers^5^ illustrates how promising this therapy is in the future detection and treatment of cancer.

**Fig. 1:**
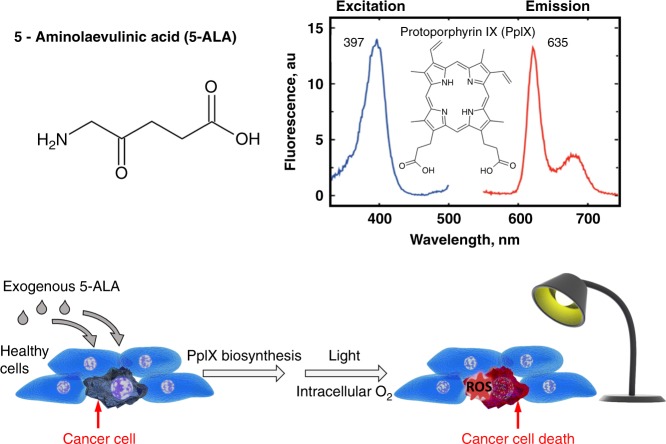
The molecular structure of 5-ALA and principle of 5-ALA-assisted photodynamic therapy in tumour cells. The spectral range of PpIX fluorescence is shown on the right; following excitation with violet blue light at 405 nm, PpIX emits a red fluorescence of 635 nm. ROS, reactive oxygen species

**Fig. 2:**
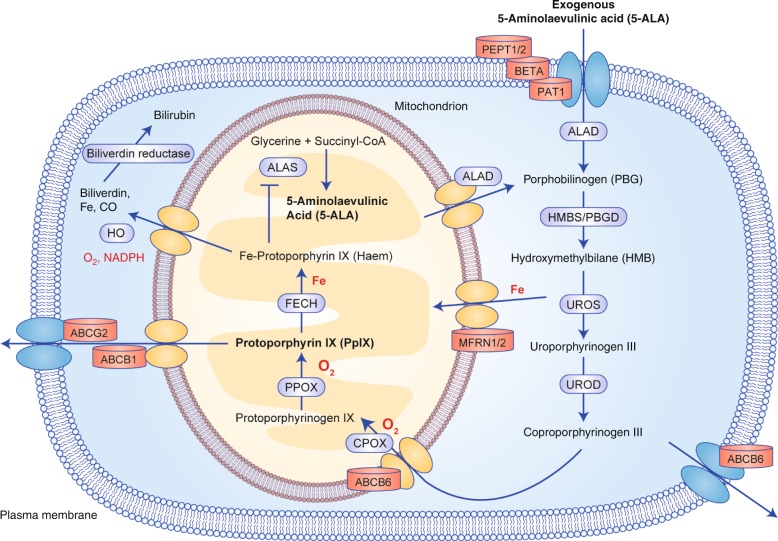
The haem synthesis pathway. ALAD 5-ALA dehydratase, HMBS hydroxymethylbilane synthase, UROS uroporphyrinogen III synthase, UROD uroporphyrinogen III decarboxylase, CPOX coproporphyrinogen III oxidase, PPOX protoporphyrinogen IX oxidase, FECH ferrochelatase, ALAS 5-ALA synthase, HO haem oxygenase, CO carbon monoxide

**Fig. 3:**
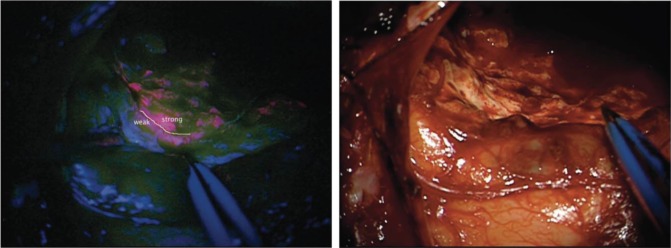
Brain tissue with intraoperative PpIX fluorescence. The left image shows the resection cavity with areas of strong (red), weak (pink separated by white line) and no fluorescence. The right image shows the corresponding white light image. Image taken from Stummer W, Tonn JC, Goetz C, Ullrich W, Stepp H, Bink A, et al. 5-Aminolevulinic acid-derived tumour fluorescence: the diagnostic accuracy of visible fluorescence qualities as corroborated by spectrometry and histology and postoperative imaging. Neurosurgery. 2014;74(3):310-319 by permission of Oxford University Press

A prodrug commonly used in both PDD and PDT is 5-aminolaevulinic acid (5-ALA), a naturally occurring amino acid (Fig. 1). 5-ALA is generated from tricarboxylic acid (TCA) cycle substrates, such as glycine and succinyl-CoA, in the first of eight steps in the biosynthesis of haem, an essential component of many critically important proteins such as cytochromes and haemoglobin, in mitochondria (Fig. 2). A negative feedback mechanism usually operates, whereby the concentration of haem regulates the production of 5-ALA, but this can be bypassed by the exogenous administration of 5-ALA to considerably boost the production of protoporphyrin IX (PpIX) in the penultimate step of this pathway. PpIX, also known as haem b or protohaem IX, is a naturally occurring photosensitiser that emits a distinctive red fluorescence following excitation with blue light (Fig. 1). Although 5-ALA is taken up by both healthy and cancer cells, 5-ALA-induced PpIX preferentially accumulates in many cancer cells and has become a useful tool for both the imaging and treatment of cancer. Fig. 3 shows human brain tumour tissue with the distinct red fluorescence of PpIX and a corresponding white light image.^6^

Since 1990, when Kennedy et al.^7^ first reported the utility of using an endogenous photosensitiser for the detection and treatment of skin cancer cells, 5-ALA/PDT has gained prominence as a non-invasive treatment option for tumours. However, the molecular mechanism(s) leading to the preferential accumulation of PpIX in cancer cells remains obscure. Tumour cells must adapt to proliferate in often harsh environments of nutrient stress and reduced pH and oxygen to fuel increased energy requirements and growth. Enhanced 5-ALA-induced fluorescence occurs in many different types of cancer, from melanomas to glioblastomas, so it is possible that metabolic reprogramming in cancer might be responsible for this phenomenon. This review looks initially at alterations in the haem pathway and transport mechanisms in tumour cells and how these alterations might explain the PpIX phenomenon, before focusing on the unique features of the tumour environment, such as altered glucose metabolism and oncogenic mutations, and exploring how this knowledge can be used in the clinical setting to improve its use in cancer diagnostics and therapies.

## Transport mechanisms and the haem synthesis pathway

### 5-ALA transporters

When 5-ALA is administered exogenously, it can move into the cell by passive diffusion through the cell membrane^8^ or by active transport through several transporters that include the peptide transporters PEPT1 (SLC15A1) and PEPT2 (SLC15A2),^9^ the amino acid transporter PAT1 (SLC36A1)^10^ and BETA transporters.^11^ Whether these transporters facilitate increased uptake of 5-ALA in tumour cells compared with normal cells is unclear. Increased levels of PEPT1 mRNA correlated with higher levels of PpIX fluorescence in renal cell carcinoma cells (Caki-1),^12^ non-small cell lung cancer cell lines^13^ and bladder cancer tissue,^14^ but no correlation between protein expression and fluorescence was seen in a study of various lines including renal cell (Caki-1), lung (A549) and bladder (T24) cancers.^15^ A small number of studies have measured the uptake kinetics of 5-ALA in tumour cells, with mixed results: no significant difference in 5-ALA uptake between healthy colonic fibroblasts (CCD18) and three colorectal cancer lines (CaCo2, HT29, SW480),^16^ but mouse fibrosarcoma (MethA) and fibroblast-like (L929) cells showed a much higher uptake of 5-ALA compared with fibroblasts (Balb/3T3).^17^ While it is not clear if cancer cells have an increased uptake of 5-ALA, knowing which transporters import 5-ALA provides opportunities for altering their expression to further enhance the accumulation of PpIX in the tumour cell.

### Transporters of PpIX and precursors

The multistep synthesis of haem from 5-ALA starts and ends in the mitochondrion (Fig. 2). In the first four steps, endogenous 5-ALA is moved into the cytoplasm where it is converted into coproporphyrinogen III, which is then moved back across the mitochondrial membrane^18^ by the ATP-binding cassette transporter ABCB6. ABCB6 can also expel coproporphyrinogen III extracellularly through the plasma membrane^19^ possibly to avoid toxic accumulation of haem intermediates as genetic defects in ABCB6 are associated with increased severity of hereditary porphyria diseases.^20^ The expression of ABCB6 has been reported to positively correlate with PpIX accumulation in murine erythroleukaemia cells (MEL)^18^ and in human glioma cells (U251, U87, T98)^21^; however, in human bladder cancer tissue with positive 5-ALA-induced fluorescence, the expression levels of ABCB6 mRNA were less than half of those measured in nearby healthy tissue.^14^ More work is needed to clarify the significance of ABCB6 as a transporter of the PpIX intermediate coproporphyrinogen III.

The efflux of PpIX is mediated via another ABC transporter, ABCG2 (also known as the breast cancer resistance protein owing to its ability to confer drug resistance through its membrane transport function).^15,22^ Not surprisingly, cell studies have shown a negative correlation between the expression of ABCG2 protein and PpIX accumulation.^15^ In human bladder cancer tissue, reduced levels of ABCG2 expression correlated with increased 5-ALA-induced PpIX fluorescence.^14^

In vitro studies manipulating the expression or activity of ABCB6 and ABCG2 transporters have successfully increased the intracellular accumulation of PpIX. Overexpression of ABCB6 in human glioma cell lines (U87,T98) significantly increased PpIX^21^ whereas inhibition of ABCG2 activity boosted PpIX fluorescence in triple negative breast cancer cells (Hs578T, MDA-MB-231)^23^ and in lung adenocarcinoma cells (A549).^15^ Therapeutic strategies that target these transporters therefore have the potential to enhance PpIX fluorescence in target cells, consequently benefitting PDD and PDT. However, a clear consistent pattern of altered flux has not been shown, suggesting that these transporters do not play a significant role in the enhanced 5-ALA-induced fluorescence seen in cancer cells.

## Alterations in the haem pathway

As the altered flux of haem precursors such as 5-ALA does not adequately explain the preferential accumulation of PpIX in tumour cells, attention is instead focused on the pathway to haem synthesis. To date, most studies looking at alterations in cancer and their impact on PpIX synthesis have focussed on the mitochondrial enzymes in the haem biosynthesis pathway, coproporphyrinogen oxidase and ferrochelatase (Fig. 2).

The enzyme coproporphyrinogen oxidase (CPOX) catalyses the oxidative decarboxylation of coproporphyrinogen III to protoporphyrinogen IX, the precursor of PpIX in the mitochondria. Adjuncts to 5-ALA PDT are reported to increase the expression of this enzyme, resulting in a boost in PpIX production. Pre-treatment of actinic keratosis in situ with adjuvant 5-fluorouracil increased protein expression of CPOX and 5-ALA induced PpIX levels in these pre-cancerous cells with significantly improved clearance rates.^24^ Increased CPOX expression has also been reported after pre-treatment with methotrexate^25,26^ and vitamin D3.^27^ These studies show the potential of adjuvants in improving the targeting and efficacy of 5-ALA therapy. Further investigation may also help to shed light on the regulation of enzymes such as CPOX and their role in the enhanced PpIX accumulation in cancer.

### A key role for ferrochelatase expression?

The last enzyme in the haem biosynthesis pathway, ferrochelatase (FECH), catalyses the insertion of ferrous ions into PpIX to form haem (Fig. 2). There is evidence of reduced FECH expression in cancer, which would be expected to result in the accumulation of PpIX. Indeed, levels of FECH mRNA are reportedly significantly lower in glioblastoma tissues,^28^ colon cancer tissues,^29^ bladder cancer tissue^14,30^ and in renal cell carcinoma cells (Caki-1)^12^ compared with healthy tissues and cells. Suppressing FECH expression by siRNA resulted in a significant increase in 5-ALA-induced PpIX in glioma cells (G112, SNB19)^28^ as well as improved PDT efficacy in four bladder cancer cell lines,^31^ which suggests that targeting FECH could be a useful adjunct to 5-ALA PDT therapy. Advances in technology, such as PDT with nanoparticles loaded with a heat shock protein siRNA, have been successful in vitro^32^ and similar technology silencing FECH has the potential to boost PpIX levels in the target tumour cell.

### Ferrochelatase suppression by microRNAs in cancer?

An obvious question is why FECH expression is repressed in some cancers. One possibility is that microRNA-mediated suppression occurs. The microRNA, miR-210, which is induced by hypoxia^33^ and highly expressed in tumours such as renal cell carcinoma,^34^ is predicted by a microRNA target prediction algorithm (TargetScanHuman, v7.1, Targetscan.org) to target FECH mRNA. However, overexpression of miR-210 significantly reduced FECH mRNA levels in rat neonatal cardiomyocytes, it did not induce a similar effect on human embryonic kidney cells (HEK-293).^35^ Whether the lowered expression of FECH seen in glioma, colon and bladder cancers is due to regulation by microRNAs, such as miR-210, needs further exploration.

### Reduced ferrochelatase activity in cancer?

Similar to reduced FECH expression, reduced FECH activity would also allow PpIX to accumulate in cells. A number of potential mechanisms might be the cause of such a reduction in FECH activity and could potentially be therapeutically exploited for use in PDD and PDT. Frataxin, a mitochondrial iron chaperone, is required for the enzyme activity of FECH .^36^ Knockdown of p53 by siRNA reduced the levels of frataxin protein and increased 5-ALA-induced PpIX more than two-fold in human embryonic kidney cells (HEK-293T).^37^ Given that mutational p53 dysfunction is common during cancer progression, a consequent reduction in the expression of frataxin could reduce the level of functional FECH and thereby contribute to PpIX accumulation in tumour cells.

FECH activity has also been shown to be inhibited by nitric oxide (NO) binding.^38^ NO is a short-lived free radical produced by nitric oxide synthase enzymes. These enzymes are highly expressed in many tumours, with complex roles in cancer progression,^39^ but whether PpIX accumulates because FECH activity is inhibited by higher levels of NO in cancer has yet to be fully explored. Co-incubation of 5-ALA with an NO donor increased the level of PpIX in prostate cancer cells (PC-3),^40^ bladder carcinoma cells (T24, 253J-P, 253 J BV) and, to a lesser extent, in healthy renal cells (RPTEC).^41^ However, NO has been shown to promote resistance to 5-ALA/PDT and even tumour persistence and progression in surviving cells,^42^ so careful consideration is needed in furthering NO as a potential PDT adjuvant.

A 2016 study found that more than 10% of small molecule kinase inhibitors clinically used in oncology have the potential to inactivate FECH, with the protoporphyrin pocket (which would normally bind to PpIX) suggested to be a binding site for these inhibitors.^43^ Interestingly, cell studies have shown that PpIX fluorescence was enhanced when 5-ALA was administered with the kinase inhibitors erlotinib^44^ and gefitinib.^45^ The effect of gefitinib was originally attributed to inhibition of ABCG2, thereby preventing PpIX efflux, but Klaeger et al.^43^ show that both inhibitors had clear interactions with FECH. In the clinic, patients with metastatic melanoma reported photosensitivity when treated with the kinase inhibitor, vemurafenib (PLX4032),^46^ which was also shown to interact with FECH in this study. The ability of inhibitors used in cancer treatment to also mediate PpIX accumulation means that they could be useful adjuncts to PDT with 5-ALA.

### Role of iron in ferrochelatase regulation in cancer

Iron deficiency is common in cancer, but whether there are reduced levels of available iron in mitochondria or a correlation with 5-ALA-induced PpIX is largely unexplored. Reducing iron availability experimentally by chelation boosted levels of PpIX in prostate cancer cells (PC-3),^40^ rat glioma stem cells (C6),^47^ squamous carcinoma cells (A431) and, to a lesser extent, in healthy lung fibroblasts (MRC-5).^48^ Breast cancer cells (MCF7) were reported to have lower levels of mitochondrial labile iron compared with healthy epithelial cells (MCF10A).^30^ In the presence of excess iron, PpIX levels dropped in the cancer cells (although they were still higher than in healthy cells), suggesting that impaired uptake of iron might be a factor in PpIX accumulation. Supporting this observation, these cancer cells expressed lower levels of mRNA for the mitochondrial iron transporters, mitoferrin-1 and mitoferrin-2.^30^ Silencing both transporters in healthy mouse fibroblasts (NIH 3T3) increased PpIX levels compared with controls.^49^ Whether the expression of the mitoferrin transporters is dysregulated in cancer is not clear. Iron transportation to FECH is suggested to be facilitated within the mitochondria by dynamic protein complexes of haem synthesis enzymes, FECH, protoporphyrinogen IX oxidase and 5-ALA synthase 2,^50^ alongside iron transporters, ABCB10 and mitoferrin-1.^51^ Perturbations in these complexes, low iron availability or impaired uptake into the mitochondria could all be contributing factors to the reduced activity of FECH and the accumulation of PpIX seen in cancers. Recent innovations exploiting this need for iron include the development of an ester prodrug that combines 5-ALA with the iron chelator, CP94.^52^ Further studies will determine whether strategies such as iron chelation will be effective in vivo for the diagnosis and treatment of cancer with 5-ALA.

## Cancer and haem synthesis

### Aberrant glucose metabolism in cancer

Porphyrias encompass a group of disorders that occur when porphyrins accumulate within the body. Acute porphyria attacks, triggered by fasting, activate haem synthesis and commonly result in the urinary excretion of excess 5-ALA and other haem synthesis precursors. Using rat and mouse models, it has been shown that induction of peroxisome proliferator-activated receptor  γ coactivator 1-α (PGC-1α), a master transcription coactivator, by the fasted liver, increased haem synthesis by upregulating hepatic 5-ALA synthase 1 (ALAS-1). Glucose intake, however, repressed PGC-1α-mediated levels of ALAS-1 in this study.^53^ Alterations in ALAS are unlikely to explain the enhanced PpIX fluorescence in tumour cells because the exogenous administration of 5-ALA circumvents this first enzyme in the haem pathway (Fig. 2). Nevertheless, this study is relevant because it does suggest that nutrient stress triggers increased haem synthesis. Nutrient stress is a feature of the harsh tumour environment to which a rapidly dividing tumour cell must adapt in order to satisfy increased demands of growth and proliferation. An early in vitro study showed that breast cancer cells (MCF7) grown under conditions of chronic glucose deprivation (0–1 mM glucose) produced higher levels of PpIX than those grown under standard conditions.^54^ Co-incubating glycolysis inhibitors with 5-ALA significantly reduced intracellular levels of PpIX in breast cancer cells (MCF7).^55^ Inhibiting glycolysis depletes ATP levels and has been shown to inactivate ABC transporters,^56^ so it is possible that a reduced flux of precursors led to this reduction in PpIX. Combining 5-ALA PDT with dichloroacetate significantly reduced cell viability in breast cancer (MCF7) cells (PpIX levels were not measured in this study).^57^ Dichloroacetate alters the flux of pyruvate in the mitochondria thereby diverting glucose metabolism from glycolysis to oxidation.^58^ These in vitro studies all suggest targeting glucose metabolism has the potential to enhance 5-ALA therapy, although further exploration is needed on the mechanistic links between altered glucose metabolism and haem synthesis in the cancer cell.

### Hypoxia and PpIX accumulation

In addition to nutrient stress and low pH, hypoxia is a common feature of the tumour microenvironment. In response to low oxygen levels, hypoxia-inducible factor (HIF) transcription factors are stabilised and alter a number of significant pathways, including haem synthesis. The expression of enzymes FECH^59^ and haem oxygenase^60^ is induced in hypoxia, suggesting an increased capacity for the conversion of PpIX into haem. Indeed, in vitro studies report that cancer cells, such as gastric cancer (KatoIII, MKN74, MKN45, TMK-1) cells,^61^ grown under hypoxic conditions have reduced 5-ALA-induced PpIX compared with those exposed to normoxia. Furthermore, silencing HIF-1α in spheroids in a three-dimensional model of colorectal cancer (SW480) increased PpIX fluorescence.^62^ Given that solid tumours are intrinsically hypoxic, strategies targeting HIF and also enhancing tumour oxygenation have the potential to improve both 5-ALA-induced fluorescence and PDT efficacy. For example, tumours in a mouse model were successfully treated with PDT using nanoparticles doubly loaded with a photochemotherapy agent and the ability to self-generate oxygen when activated by light.^63^

### NADPH and PpIX accumulation

In the haem biosynthesis pathway, the cofactor NADPH is needed for activity by the enzymes haem oxygenase^64^ and bilirubin reductase^65^ to breakdown haem into bilirubin (Fig. 2). Two recent studies suggest that aberrant NADPH production is associated with 5-ALA-induced fluorescence in glioma cells.^66,67^ NADPH is generated during the formation of α-ketoglutarate, feeding into the TCA cycle by isocitrate dehydrogenases (IDH1 and IDH2) and glutaminases (GLS and GLS2). *IDH1*-mutant human glioma cells (U87MG) with reduced levels of NADPH showed enhanced PpIX fluorescence after incubation with 5-ALA compared with their wild-type counterparts.^66^ Conversely, overexpression of *GLS2* in glioma cells (T98G, LN18, U87MG) increased the levels of NADPH but reduced 5-ALA-induced fluorescence.^67^

The question therefore arises as to whether PpIX accumulates in cancer cells because the demand for NADPH outstrips supply. Both of the studies in glioma cells show rapid consumption of NADPH after 5-ALA incubation in all cell lines, with strong PpIX fluorescence associated with the lowest levels of NADPH in glioblastoma tissue.^67^ While neither haem oxygenase activity nor haem levels were measured, the authors proposed that a reduced pool of NADPH would stall haem degradation, thereby allowing haem intermediates such as PpIX to accumulate. Although most cytosolic NADPH comes from the pentose phosphate pathway, it can also be generated by serine–glycine one-carbon (1C) metabolism by parallel pathways in the cytosol and mitochondria, the conversion of malate into pyruvate, and the TCA cycle. Given these multiple sources of NADPH, why would the pool of available NADPH be lower in cancer cells? Growth requires both energy (ATP) and biomass, and the synthesis of macromolecules such as lipids and nucleotides has been shown to consume more NADPH than ATP.^68^ Gene expression studies suggest that 1C metabolism fuels the needs of growing cancer cells. Analysis of metabolic enzyme expression across 60 cancer cell lines found that mitochondrial 1C enzymes (SHMT2, MTHFD2 and MTHFD1L) were highly expressed in rapidly proliferating cancer cells.^69^ Similar profiling of 19 cancer types identified the mitochondrial 1C enzyme MTHFD2 as the most consistently overexpressed metabolic enzyme in these tumours.^70^ Pre-treatment of cancer cells with chemotherapeutic agents methotrexate^26^ and 5-fluorouracil^71^ successfully boosted 5-ALA-induced PpIX fluorescence, and metformin enhanced the efficacy of 5-ALA/PDT.^72^ A mechanism of action common to all of these agents is blocking 1C metabolism. More work is needed to explore the effect of targeting the sources of NADPH in different cancer types on PpIX fluorescence and PDT.

### Oncogenic mutations

Oncogenic mutations alter the metabolism of the tumour cell and allow it to adapt to stresses such as nutrient deprivation. In vitro studies focussing on oncogenic mutations and their impact on the sensitivity and efficacy of 5-ALA–PDT have shown that specific mutations also promote the production of PpIX in transformed cells. Higher levels of 5-ALA-induced PpIX were reported in both breast epithelial cells (HB4a)^73^ and mouse keratinocyte cells (PAM212)^74^ transformed with *H-RAS*. Triple-negative breast cancer cells (Hs578T, MDA-MB-231) show significantly less PpIX fluorescence compared with oestrogen-receptor-positive (T47D, MDA-MB-361) and *HER2-*positive cells (SkBr3, MDA-MB-453).^23^ Similarly, *HER2* transformation of breast epithelial cells (MCF10A) doubled their PpIX fluorescence. Interestingly, the accumulated PpIX was confined to the mitochondria in the *HER2* transformed cells whereas the controls showed some membrane localisation, which rendered the transformed cells more sensitive to 5-ALA PDT.^75^ Mutations in oncogenes such as *RAS* and *MYC* promote glutamine metabolism and glutaminolysis^76^ providing TCA substrates that in turn could fuel the haem synthesis pathway. Increased expression at the protein level of most enzymes in the pathway argued for an increased capacity for haem synthesis in the *Her2* transformed cells. As FECH expression did not change, this study suggested that the accumulated PpIX resulted from the inability of FECH to keep pace with the increased flux of the haem pathway.^75^ Using gene set enrichment analysis, a cohort of acute myeloid leukaemia (AML) patients had a strong correlation between oncogene *MYCN* expression and upregulation of haem pathway enzymes.^77^

### Selective advantage to cancer cells of enhanced haem synthesis

Given the preferential accumulation of PpIX in tumour cells, it could be argued that the consequent fluorescence is a visible sign of an elevated flux of haem synthesis in cancer. It is clear that cell growth needs macromolecule synthesis fuelled by cofactors such as NADPH and using essential building blocks such as haem, and it is likely that this need is greater in a rapidly proliferating tumour. In addition, haem confers advantages on tumour progression in ways such as binding to and destabilising p53, and potentially disrupting key signalling pathways involved in tumour suppression.^78^ The haem-dependent dimerisation of progesterone-receptor membrane component 1 (PGRMC1) facilitated interactions that led to enhanced proliferation and chemoresistance in cancer cells.^79^ Haem induces the expression of haem oxygenase, which plays complex roles in the pathogenesis of cancers, with haem oxygenase expression associated with malignancy in cancers such as breast carcinoma^80^ and glioblastoma.^47^

## Conclusions

Exogenous administration of the prodrug 5-ALA significantly increases the production of the photosensitiser, PpIX, in the penultimate step of the haem synthesis pathway in mitochondria. 5-ALA-induced PpIX preferentially accumulates in many cancer cells, although a number of factors, such as alterations in haem synthesis, distinct features of the tumour environment, altered metabolism and various oncogenes, can influence the levels of PpIX, leading to corresponding changes in cell fluorescence. A summary of the possible alterations that influence the accumulation of PpIX in cancer cells, as well as their potential translational impact, is shown in Table 1.

**Table 1:** Summary of possible alterations affecting PpIX levels in cancer cells and their translational impact

Possible alterations	Effect on PpIX levels	Translational impact	Citation
*Altered haem synthesis*			
Altered flux of haem synthesis intermediates	↑	Altering activity of transporters to maximise PpIX production (includes 5-ALA uptake, influx of PpIX precursors, efflux of PpIX, iron transporters)	^12– 15, 18, 21, 23^
Increased CPOX expression/activity	↑	Pre-treatment with adjuvants (5-fluorouracil, methotrexate, vitamin D3)	^24– 27^
Reduced FECH expression/activity	↑	Targeting FECH activity at the tumour site (FECH inhibition, iron chelators, reducing influx of iron, kinase inhibitors, nitric oxide donors, Frataxin inhibition)	^12, 14, 28– 31, 37, 40, 41, 44, 45, 48, 49^
*Tumour environment*			
Hypoxia	↓	Increasing oxygen levels at the tumour site (HIF-1 inhibition, nanoparticles)	^61, 62^
*Altered metabolism*			
Altered glucose metabolism	↑	Targeting glycolysis (glycolysis inhibitors, altering glucose metabolism)	^55^
Reduced NADPH	↑	Targeting sources of NADPH (methotrexate, metformin, silencing key genes in NADPH-producing pathways)	^66, 67, 72^
*Other*			
Oncogenic mutations	↑		^23, 73– 75^

5-ALA is currently licensed by the FDA for the photodynamic treatment of dermatological conditions such as actinic keratoses, squamous and basal cell carcinomas and, more recently, in the tumour resection of high-grade gliomas.^81^ There are a number of studies and ongoing clinical trials using 5-ALA for the diagnosis and treatment of cancers including those of the bladder, female reproductive tract, prostate and brain, all of which have been extensively reviewed by van Straten et al.^5^

To overcome issues such as bioavailability and solubility, derivatives of 5-ALA have been developed (extensively reviewed by Tewari and Eggleston^82^) and shown to be successful in vivo. For example, hexyl-aminolaevulinate (HAL), a hexyl ester of 5-ALA, has been approved for endoscopic detection of bladder cancer in Europe and the USA since 2005.^3^ However, despite these improvements, 5-ALA-mediated PDT does suffer a number of significant limitations in its clinical application. The first of these is its lack of specificity. 5-ALA-induced fluorescence has been reported in vivo in non-tumour cells such as abnormal brain,^83^ multiple sclerosis^84^ and inflammatory tissue.^85,86^ It is not clear whether this non-tumour fluorescence is an inflammatory or immune response. Such examples, while rare, highlight the need to better understand the underlying mechanism of enhanced fluorescence.

Conversely, tumour cells do not always fluoresce after 5-ALA administration. In a phase II clinical trial studying 5-ALA fluorescence in high-grade gliomas, strong 5-ALA-induced fluorescence was found to be an excellent predictor of tumour presence, but over 60% of glioma samples with positive histological findings did not fluoresce.^83^ Only a small proportion of low-grade diffuse gliomas exhibited 5-ALA-induced fluorescence, although visible fluorescence was suggested to be associated with a worse prognosis.^87^ Vague, diffuse fluorescence or lack of fluorescence in brain tumours may be due to infiltrative tumour invading healthy tissue^88^ and the inability of 5-ALA to cross an intact blood-brain barrier.^89^ Strategies aimed at improving detection include combining 5-ALA PDT with sensitive quantitative fluorescent measurement,^90^ sophisticated fluorescence imaging tools such as a scanning fibre endoscope^91^ and improving light penetration.^92^

A better understanding of the mechanistic basis for the ‘vulnerability’ of tumours to the accumulation of 5-ALA should aid in the development of new approaches that improve the selectivity and sensitivity of this photosensitiser in the diagnosis and therapy of cancer. Knowing why tumour cells have enhanced fluorescence with 5-ALA might also provide insights into the basis and advantage(s) of altered metabolism in cancer. Although the molecular mechanism for the enhanced fluorescence of PpIX in cancers following 5-ALA administration is not completely defined, better exploitation of this widespread Achilles’ heel is warranted.

## Data Availability

Not applicable
